# Forecasting intraspecific changes in distribution of a wide-ranging marine predator under climate change

**DOI:** 10.1007/s00442-021-05075-7

**Published:** 2021-11-17

**Authors:** Yuri Niella, Paul Butcher, Bonnie Holmes, Adam Barnett, Robert Harcourt

**Affiliations:** 1 Department of Biological Sciences, Macquarie University, North Ryde, Sydney, NSW 2113 Australia; 2 NSW Department of Primary Industries, National Marine Science Centre, PO Box 4321, Coffs Harbour, NSW 2450 Australia; 3 Southern Cross University, National Marine Science Centre, PO Box 4321, Coffs Harbour, NSW 2450 Australia; 4 School of Science, Technology and Engineering, University of the Sunshine Coast, Sippy Downs, QLD 4556 Australia; 5 School of Biological Sciences, University of Queensland, St Lucia, Brisbane, QLD 4067 Australia; 6 College of Science and Engineering, James Cook University, Townsville, Australia; 7 Marine Data Technology Hub, James Cook University, Townsville, Australia; 8 Biopixel Oceans Foundation, Smithfield, Australia

**Keywords:** Animal telemetry, East Australian Current, Environmental correlates, *Galeocerdo cuvier*, Shark–human interaction, Species distribution model, Tiger shark

## Abstract

Globally, marine animal distributions are shifting in response to a changing climate. These shifts are usually considered at the species level, but individuals are likely to differ in how they respond to the changing conditions. Here, we investigate how movement behaviour and, therefore, redistribution, would differ by sex and maturation class in a wide-ranging marine predator. We tracked 115 tiger sharks (*Galeocerdo cuvier*) from 2002 to 2020 and forecast class-specific distributions through to 2030, including environmental factors and predicted occurrence of potential prey. Generalised Linear and Additive Models revealed that water temperature change, particularly at higher latitudes, was the factor most associated with shark movements. Females dispersed southwards during periods of warming temperatures, and while juvenile females preferred a narrow thermal range between 22 and 23 °C, adult female and juvenile male presence was correlated with either lower (< 22 °C) or higher (> 23 °C) temperatures. During La Niña, sharks moved towards higher latitudes and used shallower isobaths. Inclusion of predicted distribution of their putative prey significantly improved projections of suitable habitats for all shark classes, compared to simpler models using temperature alone. Tiger shark range off the east coast of Australia is predicted to extend ~ 3.5° south towards the east coast of Tasmania, particularly for juvenile males. Our framework highlights the importance of combining long-term movement data with multi-factor habitat projections to identify heterogeneity within species when predicting consequences of climate change. Recognising intraspecific variability will improve conservation and management strategies and help anticipate broader ecosystem consequences of species redistribution due to ocean warming.

**Supplementary Information:**

The online version contains supplementary material available at 10.1007/s00442-021-05075-7.

## Introduction

Predator foraging behaviour is a complex interplay of biological traits including physiology, morphology, predation risk and life history (Huey and Pianka 1981; Preisser et al. 2007) and is an important influence on the structure and functioning of ecological communities (Heithaus et al. 2008). While these interactions between predators and their prey are complex and occur across multiple spatial and temporal scales, they are usually centred around predators moving to locate food patches and prey moving to find resources and reduce predation risk (Lima 2002). The movements of marine animals are influenced by complex interactions of environmental and biological parameters including habitat type, depth, reproduction, individual level of site fidelity and prey availability (Speed et al. 2010; Espinoza et al. 2021). Changes in foraging behaviour occur throughout the lifespan of a predator as a consequence of ontogeny (Graeb et al. 2005) and juveniles learning how to identify particular habitat features that improve feeding success (Grecian et al. 2018). Intraspecific variation in predator behaviour can be also influenced by diet selection due to competition (Ward et al. 2006), resulting in individuals or sex classes from a population adopting different tactics to meet their energetic demands (Austin et al. 2004).

The physical properties of ecosystems also tend to have an influence on predator behaviour and habitat use. For example, by making use of high vegetation density during ambush behaviour, lions (*Panthera leo*) are more likely to successfully make a kill, even in locations with low prey abundance (Davies et al. 2016). Reduction in wind speeds can also affect predator–prey interactions by modifying species detection, locomotion and physical disturbance (Cherry and Barton 2017). In aquatic habitats, environmental gradients such as temperature, salinity, dissolved oxygen and barometric pressure, affect the patterns of space use of many predators (Block et al. 2011; Schlaff et al. 2014). Climate change is altering many of these oceanographic characteristics and influencing animal behaviour with increasing temperatures causing poleward shifts in species distributions leading to alterations in trophic structure (Vergés et al. 2016). Marine predators can also be more susceptible to fishing pressure as deoxygenation in deeper habitats compresses vertically available habitats (Vedor et al. 2021).

**Fig. 1:**
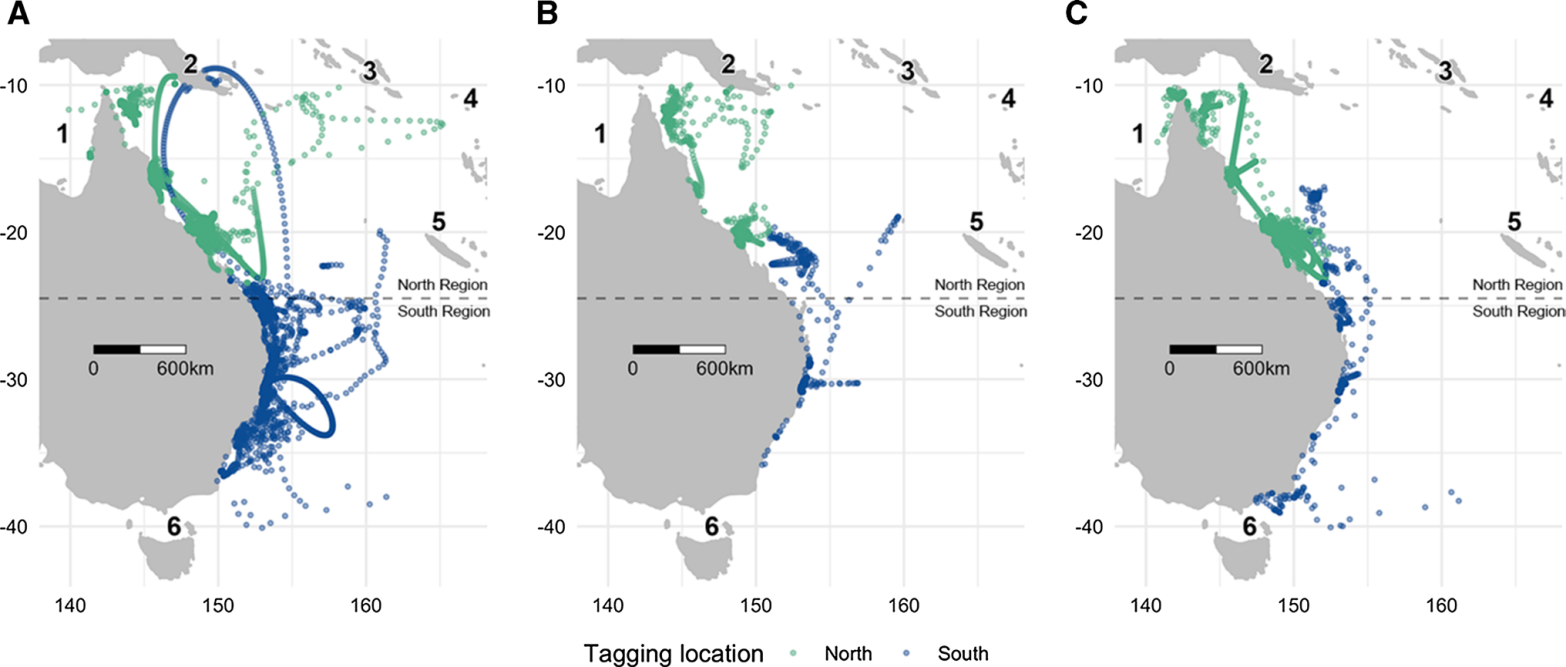
Maps of **A** juvenile female (*N* = 66), **B** juvenile male (*N* = 24), and **C** adult female (*N* = 20) daily tiger shark locations between 2002 and 2020 according to tagging location (green points = North Region; blue points = South Region). Numbers represent the (1) Gulf of Carpentaria, (2) Papua New Guinea, (3) Solomon Islands, (4) Tonga, (5) New Caledonia and (6) the Bass Straight. Horizontal dashed lines represent the coast centroid (24.5°S latitude) separating the North and the South Marine Regions

The east coast of Australia exhibits the highest rates of ocean temperature increase globally (Varela et al. 2018). This region is strongly influenced by the East Australian Current (EAC), a complex southward flowing current originating at the western boundary of the south-Pacific sub-tropical gyre (Cetina-Heredia et al. 2014). The EAC has a meandering flow that runs parallel to the coast while forming a field of mesoscale eddies and bringing nutrient-depleted warm waters down the coast (Oke et al. 2019). A strengthening of its southward component, which starts at around 31−32.5° S (Fig. 1), has been observed over the last decade (Cetina-Heredia et al. 2014). This strengthening has been responsible for several ecological disruptions including reduced foraging success of seabirds (Carroll et al. 2016), long-term heat waves causing disease outbreaks and mortality (Oliver et al. 2017), and changes in species distributions (Sunday et al. 2015; Niella et al. 2020).

The movement patterns of marine predators can vary between habitats, species, and within different individuals from the same species. Coastal movements are more sinuous when compared with more directed oceanic predator trajectories (Sequeira et al. 2018), and are often associated with seasonal shifts in prey abundance and distribution, or environmental changes such as water temperature (Speed et al. 2010). Oceanodromous species undertake long-range migrations, which may be linked to predators moving to known prey areas, or towards mating and/or pupping grounds (Sulikowski et al. 2016). Species may also exhibit partial migrations, in which a population is comprised of both migratory and resident individuals (Chapman et al. 2012). Whether climate change is leading to a convergence or a divergence in intraspecific differences in movement patterns remains to be ascertained (Galaiduk et al. 2017; Davis et al. 2020).

The tiger shark (*Galeocerdo cuvier*) is a cosmopolitan predator and exhibits many varied movement patterns (Papastamatiou et al. 2013; Lea et al. 2018). In the Atlantic Ocean, adults can traverse the western and eastern basins (Afonso et al. 2017a) and males display a higher level of philopatry than females (Lea et al. 2018). Oceanographic influences affect the movement patterns of larger migratory individuals, but these influences are less apparent for smaller, more resident sharks (Lea et al. 2018). In the Pacific Ocean, along the east coast of Australia, tiger sharks are found year-round at locations such as Raine Island on the northern Great Barrier Reef (Fitzpatrick et al. 2012), and the Chesterfield Islands in the Coral Sea region (Werry et al. 2014), but also make large-scale oceanic movements across the greater western Pacific region (Lipscombe et al. 2020). An optimal thermal regime centred around 22 °C has been proposed (Payne et al. 2018), and suggests that tiger shark dispersion and residency will change with ocean warming, as has been observed for other large coastal predators such as black marlin (*Istiompax indica*) and bull sharks (*Carcharhinus leucas*) in this region (Hill et al. 2016; Niella et al. 2020). In addition to possible shifts to stay within an optimal thermal range, tiger shark movements may also be driven or at least accentuated by potential distributional shifts in their prey due to ocean warming (Chaloupka et al. 2008; Vergés et al. 2016).

This study uses almost 2 decades of acoustic and satellite tracking data to identify sex and maturation class differences in movement patterns of tiger sharks off the east coast of Australia and predict the future species distribution over the next decade. Through the application of both traditional and new spatial modelling approaches, and incorporating observed and forecasted oceanographic and biological (i.e. predicted distributions of potential prey species) factors that likely influence tiger shark movement, our approach identifies distributional responses among biological classes arising from intraspecific variation in movement patterns. Understanding class-specific differences in long-term redistribution patterns of this marine top predator will help anticipate broader ecosystemic impacts along the east coast of Australia as a consequence of ocean warming. Our approach could be applied to obtain more accurate predictions of marine predator redistributions in response to climate change, by accounting for both intraspecific variation in movement and changes to potential prey occurrence.

## Materials and methods

### Shark tracking

Tiger sharks were tagged along the entire east coast of Australia (Fig. S1) between 21 February 2002 and 7 January 2020 (Table S1). Sharks were captured and tagged as outlined in Fitzpatrick et al. (2012) and Holmes et al. (2014). A total of 115 tiger sharks (86 females, 29 males) ranging from 150 to 386 cm total length (TL) (Fig. S1) were tracked off the east coast of Australia between 21 February 2002 and 29 March 2020 (Table S1; Fig. S1). Eighty-four sharks were fitted either internally or externally with acoustic transmitters (V16; VEMCO), 66 with satellite transmitters consisting of 48 Smart Position and Temperature (SPOT; Wildlife Computers) attached to the first dorsal fin and 18 Pop-up Satellite Archival Tags (PSAT; both miniPat and mk10 models; Wildlife Computers) attached to the dorsal musculature at the base of the first dorsal fin. Thirty-five animals were tagged with both acoustic and satellite transmitters (Fig. S1). Acoustic locations were obtained from 108 receivers deployed between 36.91° S, 146.49° E and 18.59° S, 153.71° E (i.e. ~ 2150 km stretch of coastline) from 27 May 2015 to 21 January 2020 (Fig. S1). Receivers included those deployed by NSW Government research programmes and the Integrated Marine Observing System (IMOS) Animal Tracking network (Hoenner et al. 2018). Quality control was performed by identifying and excluding any false detections prior to analysis. A complete description of the shark location processing is included in the Appendix S1.

### Environmental variables

Daily remote sensing data on sea surface temperature (SST) and chlorophyll-a concentration with a 0.2° resolution were downloaded from IMOS through the Australian Ocean Data Network portal (https://portal.aodn.org.au/; Accessed 05 March 2020). Data were available from 5 July 2002 onwards and obtained for the entire area where tiger sharks were tracked during the study period, spanning 40.40° S, 153.60° E and 7.00° S, 167.40° E. The analyses were conducted using a grid with environmental data aggregated at a 0.5° latitude × 0.5° longitude daily resolution. Shark locations were matched to the grids of environmental data and daily values for each variable obtained for the corresponding grid cell on the previous 12 days. To overcome issues with missing data due to daily cloud coverage and account for possible lag times in environmental factors affecting movement, 6-day averages were calculated for each environmental variable by first splitting the 12-day windows in half. These values were used to obtain derivatives (i.e. subtracting the average of the second half of the 12-d window from the first half of the 12-d window) for each variable, to investigate how different biological classes responded to changes in environmental parameters. As east Australian tiger sharks show a thermal preference around 22 °C (Payne et al. 2018), a second SST variable was also tested (hereafter referred to as SST optimal), an SST derivative from the 22 °C isotherm using the same 6-day resolution (Table S2).

The monthly Oceanic Niño Index (ONI) was included in the analysis. This scale ranges from negative to positive (i.e. cooling to warming periods) values, based on a threshold of ± 0.5 °C of 3-month means from the Extended Reconstructed Sea Surface Temperature (ERSST) version five (https://origin.cpc.ncep.noaa.gov/products/analysis_monitoring/ensostuff/ONI_v5.php; Accessed 20 April 2020), calculated for the Niño 3.4 region (from 5° N, 120° W to 5° S, 170° W). Thereby, a La Niña month would have an ONI value lower than − 0.5, and El Niño month have ONI values higher than 0.5 (Fig. S2).

### Modelling framework to predict intraspecific variation in shark distribution

Future tiger shark distribution along the east coast of Australia was assessed by first identifying class-specific patterns in dispersal, habitat preferences and vertical movements within this region (Class-specific patterns in shark dispersalandhabitat preference), and then forecasting the distribution of each biological class according to the relative influence of environmental and biological factors (2.5). Tiger sharks were divided into four classes using sex-specific maturation sizes for the east coast of Australia (Holmes et al. 2015): (1) juvenile females: females < 326 cm total length (TL); (2) mature females: females ≥ 326 cm TL; (3) juvenile males: males < 297 cm TL; (4) mature males: males ≥ 297 cm TL.

The east coast of Australia has two main bioregions (https://www.waterquality.gov.au/anz-guidelines/your-location/australia-marine-regions; Accessed 17 April 2020), the northern Coral Sea Marine Region, including the Great Barrier Reef Marine Park; and the Temperate East Marine Region to the south (Fig. S1). These two main areas intersect around the 24.5°S latitude (Fig. S1), so this location was considered a suitable divisor at which to compare tiger shark movements within the North (i.e. location latitudes < 24.5°S) versus the South (i.e. location latitudes ≧ 24.5°S) Marine Regions. The habitat across the study area ranges from warm tropical Great Barrier Reef waters in the north through a sub-tropical and warm temperate mixture of coral and rocky reef ecosystems further south. Therefore, shark habitat preferences were modelled independently for the North and South Regions to ascertain the specific drivers of shark presence within each zone.

### Class-specific patterns in shark dispersal and habitat preference

Both satellite and acoustic locations were combined to investigate tiger shark latitudinal movements. Depth data from PSATs were used to assess possible temporal and environmental changes in shark vertical movements. For this purpose, available maximum dive depths were matched to the daily locations of the tracks for each individual. All satellite tracks were then used to investigate fine-scale movement patterns. First pseudo-absences of the satellite-tracked individuals were estimated to identify where they could have gone given average speed and duration, computing 100 simulated tracks for each shark starting from the original tagging locations and using observed distributions of turning angles and step lengths from the tracks. Simulated tracks were constrained to the minimum convex polygon of the real track and a land boundary was added to restrict positions to in-water. Only the first 45 simulated tracks from each shark were used in the modelling as this value was found to stabilise all environmental variables (Fig. S2) (Payne et al. 2018). The location data were classified into the North and South Regions according to the respective track latitudes in relation to the 24.5°S coast centroid.

All statistical analyses were performed in R (version 4.0.5). Movement patterns were assessed using Generalised Additive Mixed Models (GAMM) with the mcgv R package (Wood 2017). Adult males (*N* = 5) were excluded because of the small sample size (Table S1). To account for pseudo-replication and reduced spatial and temporal autocorrelation, only consecutive daily locations separated by 0.25° were included in the modelling (Lea et al. 2018). Models included shark identification number (ID) as an intercept, tagging location (i.e. North or South Region), and year as random effects, to account for individual variations in movement patterns and the unbalanced nature of the annual sampling.

The candidate predictors tested included the environmental variables ONI, the derivatives of SST and chlorophyll-a, SST optimal, as well as month (Table S2). Multicollinearity was assessed with Pearson’s correlations, and since no predictors were found to be significantly correlated, they were all included in the analysis. To investigate possible class-specific responses to the candidate predictors tested, the three biological classes were included as interacting effects with all explanatory variables, except in the vertical model. Maximum number of degrees of freedom for smooth functions (*k*) was limited to 10 for all interacting effects to avoid model overfitting. Variables were gradually added in a stepwise manner and only retained if they led to a significant improvement in model fit. Final models were selected based on higher AIC weights and visually inspected for a normal residual distribution.

#### Model 1: latitudinal movements

Shark dispersion was modelled with observed latitude from both acoustic and satellite transmitters as the response variable (Table S2), and a Gaussian family error distribution.

#### Model 2a: North Region occurrence and Model 2b: South Region occurrence

Tiger shark occurrence was modelled independently for the North and South Regions and included presence (i.e. one value, attributed to the real tracks) and absence (i.e. zero value, attributed to the simulated tracks) data (Table S2), using Binomial families of error distribution. Tiger sharks tagged in the North Region were not observed to move into the South Region, therefore, tagging location was not included as a random effect for the South Region occurrence model.

#### Model 3: vertical movements

Due to the limited number of sharks with good quality (i.e. more than 10 days of data) depth data (*N* = 21), patterns in vertical movements were pooled at the species level. This vertical model included the corresponding individual daily maximum dive depths as the response variable (Table S2), thus analysed with a Gamma family error distribution. In the vertical model, interactions were tested between the variables Marine Region (i.e. North or South, classified based on track locations) and month, and between Marine Region and ONI, to respectively account for possible spatial differences in temporal and environmental trends of shark vertical movements.

### Predicting 2021–2030 shark habitat suitability

Multifactor habitat suitability models have been found to be more efficient than simpler temperature-based models to explain species distribution patterns (McHenry et al. 2019). Projections of marine species distributions have mostly aimed at finding the temperature gradients correlated with a species occurrence, followed by the use of forecast water temperatures to identify the areas where animals might be found in the future. Redistribution of large migratory marine predators is also likely to be influenced by changes in occurrence of their potential prey.

#### Base models: biological and environmental variables

Data on occurrence of potential tiger shark prey, i.e. sea turtles, crabs, sea snakes, dugongs, birds, teleosts and elasmobranchs (Simpfendorfer et al. 2001; Ferreira et al. 2017), between January 2002 and December 2020 were downloaded at the species level from the Ocean Biodiversity Information System (https://mapper.obis.org/; Accessed on 13 May 2021), with a resolution of 0.5° for the entire east coast of Australia (Table S7) and log-transformed (Fig. S4). Tiger shark location data were used to create class-specific occurrence matrices for the 2002–2020 period. In each matrix, grid values corresponded to the total number of class-specific shark locations observed within each cell. Relationships between predator and prey occurrence were assessed independently for each shark biological class using Generalised Linear Models (GLM), including number of shark locations as the response variables with Poisson families of error distributions, and species-specific potential prey occurrences as candidate predictors.

The daily SST data between 2002 and 2020 were aggregated monthly and then used to calculate respective yearly mean values. Yearly values were then averaged to obtain a proxy of the 2002–2020 water temperature distribution across the study area, and this was matched to the class-specific matrices of shark presence and each potential prey species. Our approach takes into consideration that not only predators, but also their prey, will move with temperature changes. Therefore, temperature habitat suitability models (hereafter referred as the base models) were calculated for each shark biological class and their corresponding significant sets of potential prey species (identified from the GLMs) using Generalised Additive Models (GAM), respectively, including number of shark locations (Poisson distribution) and the log-transformed potential prey occurrence (Gamma distribution) as the response variables. When potential prey species distributions were not significantly influenced by temperature variation, those species were excluded from the analysis.

**Fig. 2:**
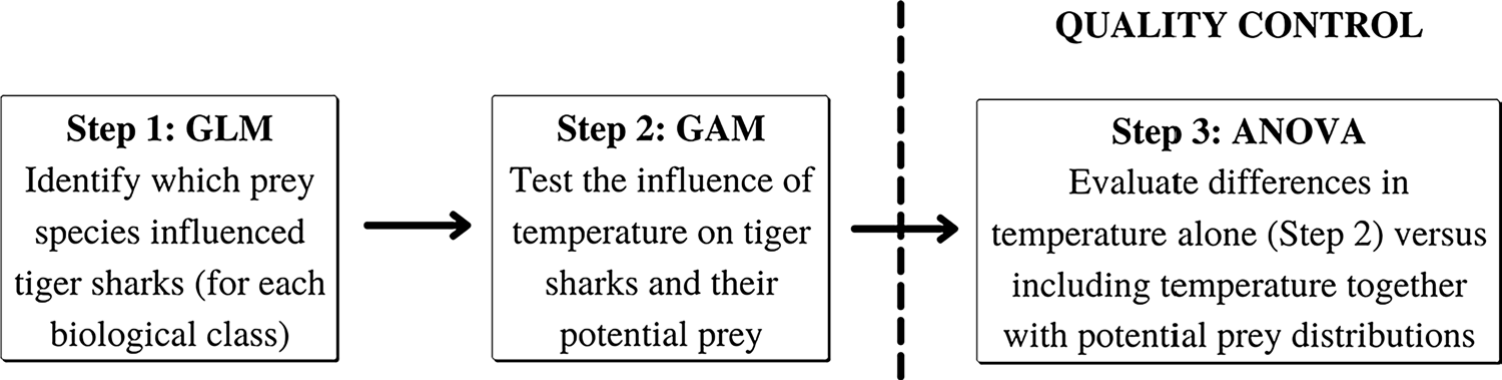
Flow chart of the predictive modelling calibration process including Generalised Linear Models (GLM), Generalised Additive Models (GAM) and Analysis of Variance (ANOVA)

A preliminary approach was conducted to assess the significance of including potential prey distribution data to forecast the class-specific distributions of tiger sharks (Fig. 2). Model performances were compared using temperature alone (partial model = temperature base model) and temperature together with the observed distributions of potential prey that were influenced by temperature (full model), during the 2002–2020 period. Partial and full models (i.e. GAMs) were calculated independently for each shark biological class and their corresponding sets of positively associated potential prey species, and compared using an Analysis of Variance (ANOVA) (Fig. 2).

#### Predictive models: class-specific habitat suitability

Since partial models were found to significantly explain (ANOVA *p*-values < 0.001) lower percentages of deviance (Juvenile females = 4.1%; Juvenile males = 13.7%; Adult females = 17.6%) than full models (Juvenile females = 44.5%; Juvenile males = 23.3%; Adult females = 48.3%), predictive models included both shark and potential prey redistribution layers. Daily predicted SST data with 0.25° resolution was downloaded from the Coupled Model Intercomparison Project (CMIP) 6 of the World Climate Research Programme (available at https://esgf-node.llnl.gov/search/input4mips; downloaded 20 January 2021) for the period 1 January 2021 to 31 December 2030 and aggregated monthly at the same spatial resolutions of the potential prey occurrence data. These monthly layers were used to run temperature models for each combination of potential prey species, which together with temperature data were fed into the full models. Projections of thermal habitat suitability were then aggregated by shark class and their corresponding groups of potential prey, and then averaged for every future year. All yearly layers were then averaged to obtain class-specific predicted models of tiger shark suitable habitats to 2030.

## Results

Data from 66 juvenile females (average track length = 221 days), 24 juvenile males (296 days) and 20 adult females (308 days) were used (Table 1). Most juveniles travelled total distances of up to 1418 (males) and 2395 (females) km, whereas adult females travelled shorter maximum distances of 1350 km (Table 1). Sharks moved along the entire east coast of Australia from the Bass Straight to the Gulf of Carpentaria, and towards Papua New Guinea, the Solomon Islands, Tonga, and New Caledonia (Fig. 1; Appendix 1). While sharks tagged in the North Region remained within this region, some individuals tagged in the South Region were observed to move into the North Region (Fig. 1).

**Table 1:** Tracking summary of 90 juvenile and 20 adult tiger sharks monitored between 2002 and 2020 along the east coast of Australia, grouped according to their corresponding biological class (Class) and tagging locations (North and South Regions)

Class	Tagging location	Female								Male							
		*N*	TL	Tracking days			Distance travelled			*N*	TL	Tracking days			Distance travelled		
				Min	Mean	Max	Min	Mean	Max			Min	Mean	Max	Min	Mean	Max
Juvenile	North	18	282.1 ± 33.5	16	286	1300	28.4	519.5	2137.8	6	258.2 ± 27.7	94	242	409	79.4	408.3	927.9
	South	48	227.5 ± 45.0	4	157	948	2.8	508.1	2394.8	18	226.3 ± 40.3	2	145	651	9.9	344.6	1418.2
Adult	North	13	348.6 ± 19.1	17	192	472	64.8	350.4	1164.0								
	South	7	345.4 ± 13.6	15	425	1580	260.0	751.7	1350.2								

Included are the respective number of sharks tagged (*N*), mean (± SD) total length cm (TL), number of tracking days, and distances travelled (km) per each sex

### Shark dispersal, habitat preference and vertical movements

**Fig. 3:**
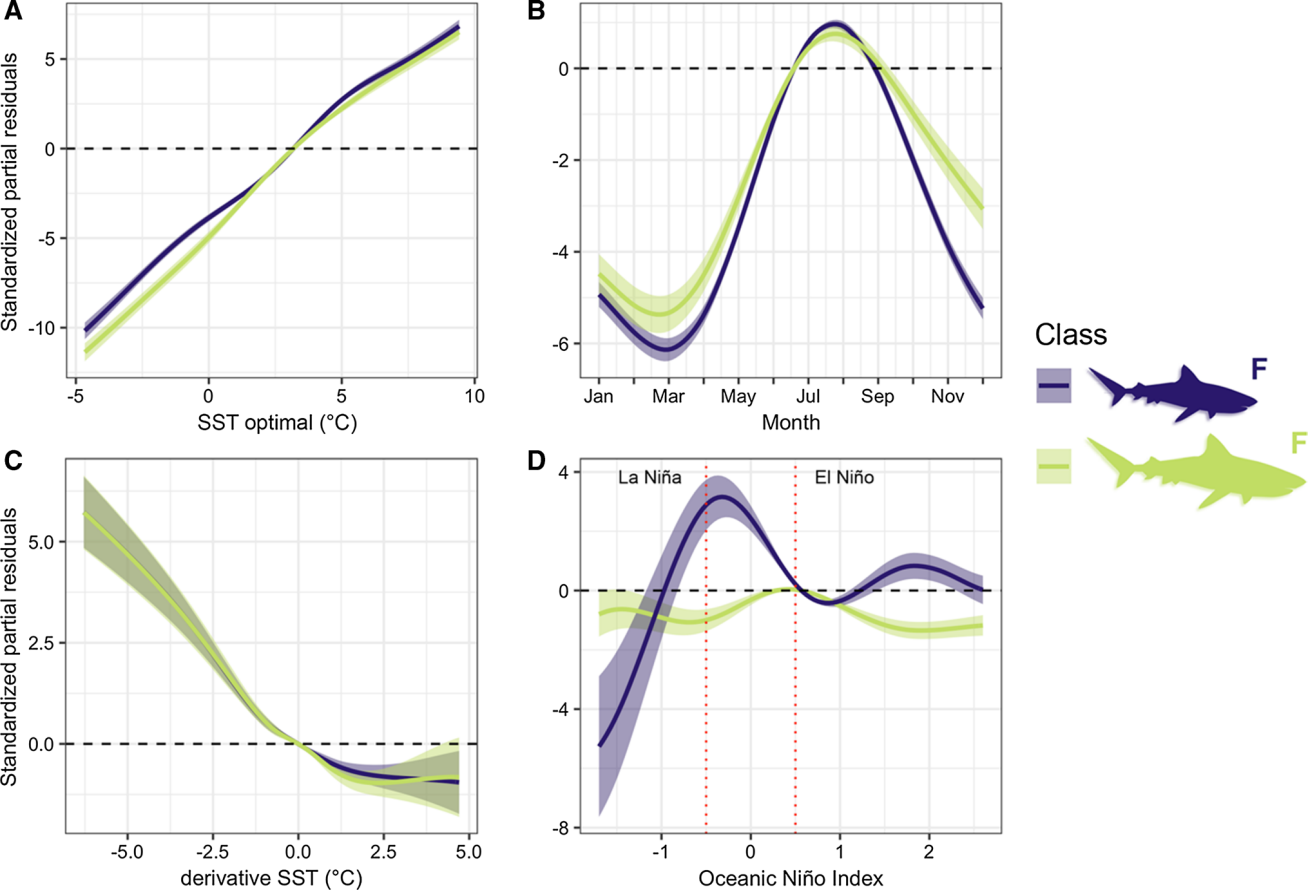
Generalised Additive Mixed Model of tiger shark latitude along the east coast of Australia (Model 1), including the significant effects of **A** sea surface temperature (SST) optimal, **B** month, **C** derivative SST, and **D** Oceanic Niño Index. Shaded areas and horizontal dashed lines represent the 95% confidence intervals and null effects, respectively. Red dotted lines (**D**) represent the 0.5 thresholds characterising La Niña (negative) and El Niño (positive) events. The colour legend represents the significant interacting biological classes of juvenile females (F) (*N* = 66) and adult females (F) (*N* = 20)

Female tiger shark southward dispersal was influenced by periods of warming temperatures, with effects observed for shark ID, tagging location and year (Table 2; Fig. 3).

**Table 2:** Generalised Additive Mixed Model of tiger shark latitudinal movement

Type	Variable	edf	Ref.df	*F*	*p*	Dev.exp
Fixed						79.5%
	Class x SST optimal	8.63	8.97	593.60	< 0.001	
	Class x month	7.96	7.99	491.00	< 0.001	
	Class x derivative SST	6.98	7.78	66.95	< 0.001	
	Class x Oceanic Niño Index	7.89	7.99	31.26	< 0.001	
Random						13.4%
	Shark ID	76.74	82.00	243.20	< 0.001	
	Tagging location	0.99	1.00	2329.00	< 0.001	
	Year	0.01	1.00	< 0.01	< 0.001	

Included are the effective degrees of freedom (edf), reference degrees of freedom (Ref.df), *F*-value (*F*) and *p* value (*p*) of each model variable, and the respective percentages of deviance explained (Dev.exp) from fixed and random effect variables (Type)

While juveniles had northward dispersal during weak La Niña, both classes tended to move southwards during stronger La Niña periods (Fig. 3). Opposing trends of northward and southward movements were observed for juvenile and adult sharks, respectively, during El Niño (Fig. 3).

**Fig. 4:**
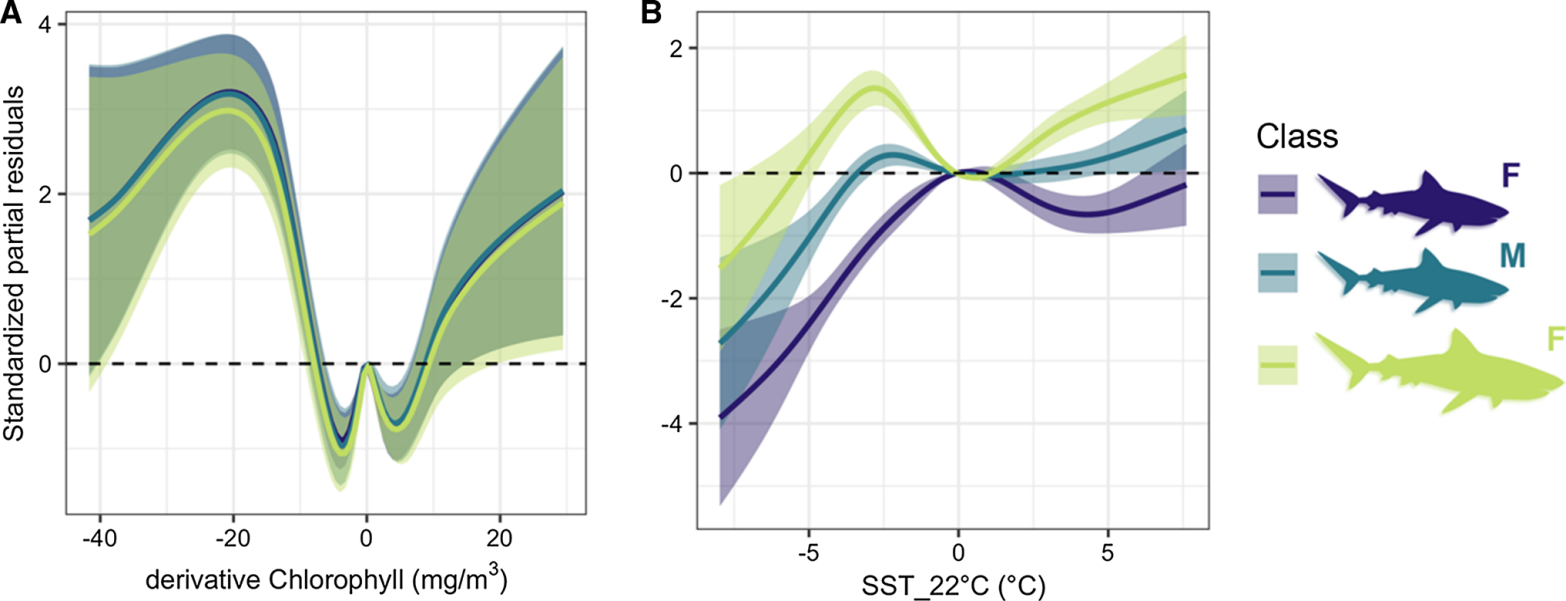
Generalised Additive Mixed Models of tiger shark occurrence at the (**A**) North (Model 2a) and (**B**) South (Model 2b) Regions, including the respective significant effects of **A** derivative chlorophyll-a and **B** sea surface temperature (SST) difference from the 22 °C isotherm. Shaded areas and horizontal dashed lines represent the 95% confidence intervals and null effects, respectively. The colour legend represents the significant interacting biological classes of juvenile females (F) (*N* = 66), juvenile males (M) (*N* = 24) and adult females (F) (*N* = 20)

The occurrence models indicated that tiger shark habitat preferences in the North and South Regions were influenced by different environmental factors, i.e. the derivatives chlorophyll-a concentration and optimal SST, respectively (Table 3). While shark habitat preferences in the North Region did not vary among individuals tagged at different locations, both regions showed significant inter-individual and yearly variation (Table 3). The presence of all three tiger shark classes in the North Region were associated with larger changes in chlorophyll-a concentration (Fig. 4a), while patterns in the South Region were associated with class-specific water temperature gradients from the optimal SST (Fig. 4b). While juvenile females present in the South Region associated with waters between 22 and 23 °C, presence of adult females and juvenile males was predominantly related with cooler (i.e. 18–22 °C and 19–22 °C, respectively) or warmer (i.e. > 23 °C for adults) temperatures (Fig. 4c). While these relationships explained very little of the variability in the data (Table 3), the effect of optimal SST upon tiger shark presence in the South Region was much more pronounced (3.5%) than that of chlorophyll-a in the North Region (0.2%).

**Table 3:** Generalised Additive Mixed Models of tiger shark habitat preference in the North and South Regions

Region	Type	Variable	edf	Ref.df	*F*	*p*	Dev.exp
North	Fixed						0.2%
		Class x derivative chlorophyll-a	8.08	8.61	125.00	< 0.001	
	Random						3.2%
		Shark ID	46.67	50.00	2106.00	< 0.001	
		Tagging location	< 0.01	1.00	< 0.01	0.560	
		Year	< 0.01	1.00	< 0.01	< 0.001	
South	Fixed						3.5%
		Class x SST optimal	8.87	8.99	331.90	< 0.001	
	Random						3.1%
		Shark ID	0.18	20.00	352.40	< 0.001	
		Year	< 0.01	1.00	< 0.01	0.022	

Included are the effective degrees of freedom (edf), reference degrees of freedom (Ref.df), *F*-value (*F*) and *p* value (*p*) of each model variable, and the respective percentages of deviance explained (Dev.exp) from fixed and random effect variables (Type)

**Fig. 5:**
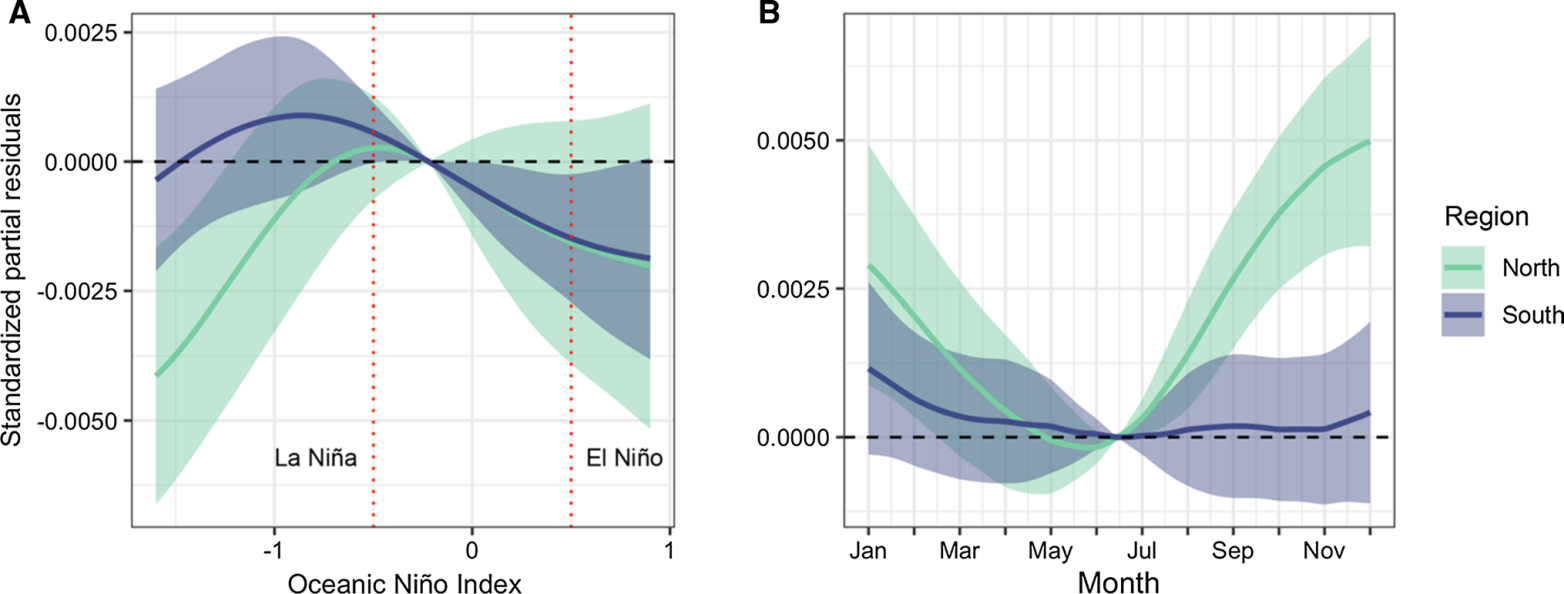
Generalised Additive Mixed Model of tiger shark maximum dive depths (*N* = 21) along the east coast of Australia (Model 3), including the significant effects of **A** Oceanic Niño Index, and **B** month. Shaded areas and horizontal dashed lines represent the 95% confidence intervals and null effects, respectively. Red dotted lines (**A**) represent the 0.5 thresholds characterising La Niña (negative) and El Niño (positive) events. The colour legend represents the significant interacting marine regions

Maximum dive depths in the North and South Region did not vary significantly across years, tagging location or individuals tracked, but were influenced by ONI and month (Table 4). Tiger sharks made use of shallower isobaths in the North Region during stronger La Niña periods and during El Niño they made shallower dives in the South Region (Fig. 5a). Sharks used deeper waters in the North Region during the austral spring and summer, but no clear seasonal vertical patterns were observed in the South Region (Fig. 5b).

**Table 4:** Generalised Additive Mixed Models of tiger shark maximum dive depths

Type	Variable	edf	Ref.df	*F*	*p*	Dev.exp
Fixed						19.3%
	Marine Region x ONI	6.23	7.35	3.39	< 0.001	
	Marine Region x month	7.11	7.69	5.57	< 0.001	
Random						28.8%
	Shark ID	0.17	19.00	20.98	0.156	
	Tagging location	< 0.01	1.00	< 0.01	0.936	
	Year	3.05	8.00	53.41	0.172	

Included are the effective degrees of freedom (edf), reference degrees of freedom (Ref.df), *F*-value (*F*) and *p* value (*p*) of each model variable, and the respective percentages of deviance explained (Dev.exp) from fixed and random effect variables (Type)

### Predicting 2021–2030 shark habitat suitability

The descriptive models (Shark dispersal, habitat preference and vertical movements) point to periods of warming temperatures leading to female tiger sharks moving towards higher latitudes (i.e. into the South Region), where the presence of the different biological classes were correlated with temperature gradients. This supports the inclusion of water temperature in predicting tiger shark redistribution with climate change. Occurrence of each shark biological class was found to be significantly correlated with different sets of potential prey species, but all selected for the sea turtle, bird and teleost groups (Table S8), corroborating the hypothesis that predator movements will be linked with the distributions of potential prey. The influence of thermal habitats upon the distributions of potential prey between 2002 and 2020 were not significant for hawksbill turtles (*Eretmochelys imbricata*), olive sea snakes (*Aipysurus laevis*) nor spotted eagle rays (*Aetobatus ocellatus*) (Table S9), so these species were removed from the analysis.

**Fig. 6:**
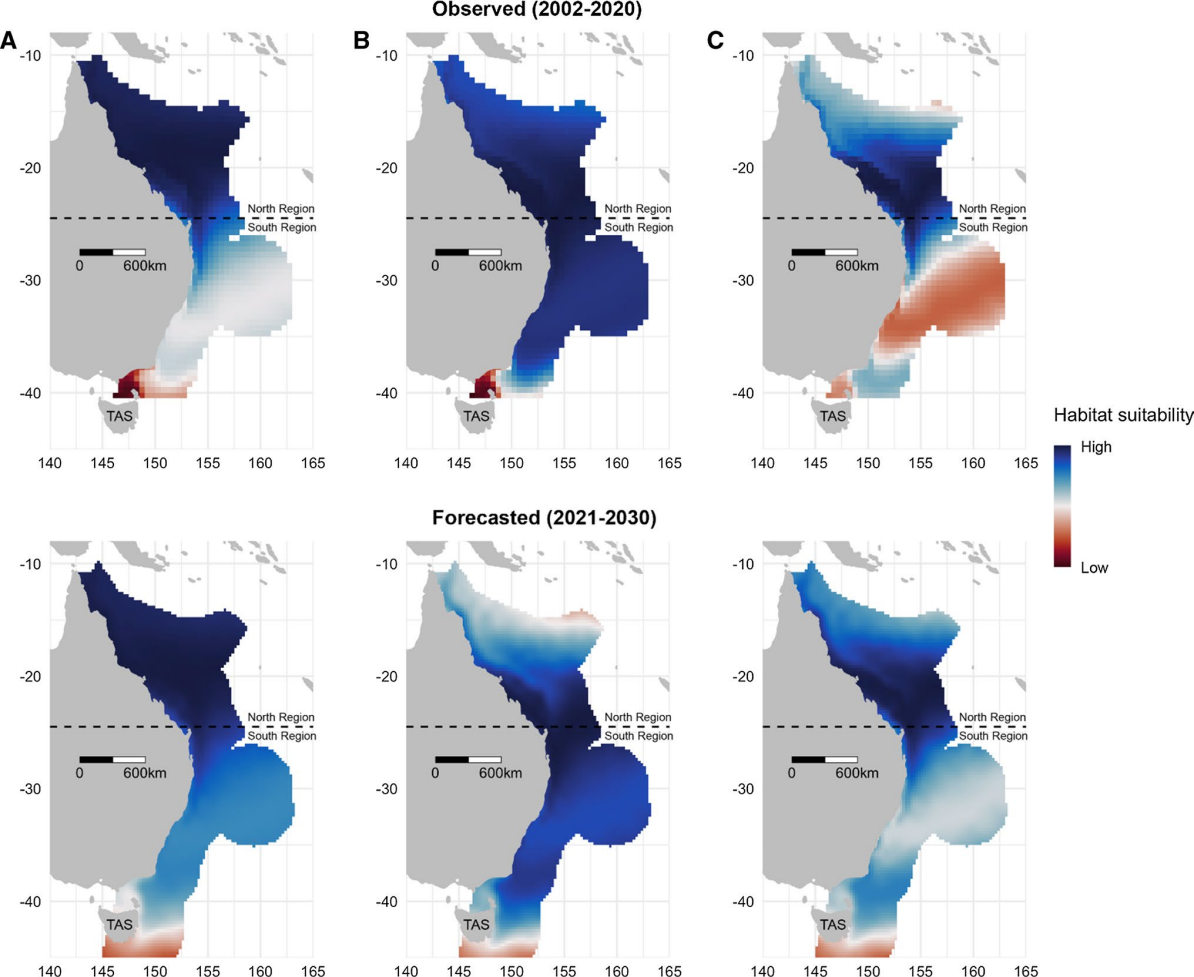
Observed (upper panel) and forecasted (lower panel) habitat suitability models (Model 4) for **A** juvenile female, **B** juvenile male, and **C** adult female tiger sharks for the next decade to 2030. Horizontal dashed lines represent the coast centroid (− 24.5° latitude) separating the North and the South Marine Regions (*TAS* Tasmania)

Over the next decade, these models predicted that tiger shark suitable habitats will shift south extending from ~ 40°S towards the east coast of Tasmania (~ 43.5°S) with some differences between each biological class (Fig. 6). Female tiger sharks are forecast to remain primarily in the North Region by 2030 but their intrusions into the South Region are expected to become more frequent, particularly for juveniles (Fig. 6a and c). On the other hand, while juvenile males had a broad distribution along the entire east coast of Australia during the 2002–2020 period, they are forecast to become less frequent in the North Region and to make more prominent use of the South Region by 2030 (Fig. 6b).

## Discussion

Intraspecific differences in movement patterns can be driven by distinct dietary needs of each biological class and competition for resources, which in turn is affected by resource distribution in space and time, and the abilities of the competing classes (Ward et al. 2006). Tiger sharks along the east coast of Australia exhibited distinct intraspecific variation in movement patterns that differ between northern tropical and southern temperate bioregions. Individuals tagged in the north tended to remain local, but individuals tagged in the south moved more broadly, similar to, but over a much broader geographical scale, than reported elsewhere for example off Hawaii (Papastamatiou et al. 2013). The high percentages of deviance, between 23.3 and 48.3%, explained by the predicted habitat suitability models that included the distribution of potential prey, suggest that tiger shark movements are in part driven by foraging. Redistribution of class-specific groups of potential prey, such as the ones found here due to ocean warming (Table S9), may exacerbate intraspecific differences in the future distributions of marine predator species.

Climate change is rapidly altering the structure and functioning of temperate coastal areas across different trophic levels and identifying the environmental and biological drivers of redistribution is paramount to forecasting where species will be found in the future. With warming waters, marine predators are moving poleward, including southward shifts associated with the strengthening of the East Australian Current (Hill et al. 2016; Niella et al. 2020). While water temperature is a key factor affecting species redistributions, here, we found that different biological classes can respond in distinct ways. While similar patterns of southward female dispersal were observed during warming months, juveniles moved northwards and adult sharks moved southwards during stronger El Niños, which have stronger effects at higher latitudes (Holbrook et al. 2009). Climate change is expected to reduce the swimming performance of juvenile ectothermic elasmobranchs confined to nursery habitats, while physiological capabilities could allow wide-ranging species such as the tiger shark to explore habitats not available to others (Lear et al. 2019). Our predictive model pointed to broader range expansion for the juvenile male class. Since tiger sharks are wide-ranging from an early age (Lea et al. 2018), juvenile individuals may be somewhat resilient to ocean warming as they have the capacity to move to new areas (Payne et al. 2018). Impacts of climate change upon primary producers include the disappearance of kelp caused by a rise in activity of herbivorous fish moving southwards (Vergés et al. 2016). The increased presence of top predators at higher latitudes in future years, such as that predicted in this study, might help control the populations of shifting lower trophic level species, potentially buffering ecosystem impacts from ocean warming through top–down effects.

For two or more species with high overlapping niches to coexist they need to have similar fitness and lower levels of interspecific than intraspecific competition (Chesson and Kuang 2008). In the North Region, tiger sharks may occupy higher trophic levels than in the South Region (Ferreira et al. 2015), in part explaining why female tiger sharks are expected to remain mostly within lower latitudes by 2030, as they may benefit from richer prey sources there. By contrast, juvenile males might be less competitive with the female classes, and therefore, could be favoured to follow the shifts of temperature and potential prey distribution towards higher latitudes. The predicted higher tiger shark presence at higher latitudes over the next decade may potentially lead to an increase in interspecific competition as range overlap might increase with white (*Carcharodon carcharias*) (Spaet et al. 2020; Lee et al. 2021), bull (Lee et al. 2019) and sevengill (*Notorynchus cepedianus*) sharks (Barnett et al. 2010, 2017). Since temperature also influences the movements of these other sharks, they are also likely to be undergoing distribution shifts along the east coast of Australia (Niella et al. 2020). Overlaps between these species will thus be influenced by whether they will converge or not to occur in the same areas and share similar resources. For example, tiger and bull sharks are known to co-occur in other regions (Afonso et al. 2017b), but to have distinct depth preferences leading to each species targeting pelagic or coastal prey (Trystram et al. 2017; Niella et al. 2021). Further research on resource partitioning is needed to better understand how sympatric predators will coexist as the oceans warm.

The differences in movement patterns of juvenile and adult female tiger sharks may be not only linked to thermal preferences, but also to individuals learning to take advantage of particular areas during periods of increased prey abundance. Some tiger sharks might specialise on particular prey (Matich et al. 2011) and appear capable of returning to specific locations when there are seasonal increases in prey abundance such as fledging albatross, *Phoebastria *spp. (Meyer et al. 2010) or turtles at Raine Island (Fitzpatrick et al. 2012). The densities of these prey can be affected by water thermal gradients as seen in our 2002–2020 analysis, and by El Niño events, with a decrease in seabird populations in the southern Great Barrier Reef due to reduced food availability (Devney et al. 2009), and increased green turtle recruitment (Limpus and Nicholls 1988; Dunstan et al. 2020). Our predictive modelling framework accounts for such environmental factors influencing the redistributions of potential prey species to forecast the preferred habitats of each marine predator biological class over the next decade.

Sharks have a variety of vertical movements including diel migrations, oscillatory vertical displacements, and both surface or bottom swimming modes, which are related to foraging or navigation purposes (Speed et al. 2010; Andrzejaczek et al. 2019). Tiger sharks were found to use shallower waters in the South Region during El Niño periods, when there is southward displacement of the EAC flow particularly towards 37°S (Cetina-Heredia et al. 2014). This trend is contradictory to what would have been expected if tiger sharks were experiencing vertical niche expansion as a consequence of periods of intensified warmer waters at higher latitudes. However, this hypothesis remains speculative due to our limited sample size. The use of deeper isobaths from the North Region during the austral summer could be linked to individuals escaping hotter surface waters but is more likely linked to tropical submergence while sharks are travelling southwards (Carlson et al. 2010; Niella et al. 2017).

Understanding how marine animals move as seas warm is needed to anticipate potential broad ecosystem impacts from climate change. Large-bodied animals might be less vulnerable to changes in thermal habitat availability as they can adapt to different areas (Bangley et al. 2018; Payne et al. 2018; Niella et al. 2020). Tiger sharks from the east coast of Australia showed individual variation in movement patterns with age, habitat type and geographical location influencing patterns of space use. This intraspecific variation, both within and between biological classes, suggests an uneven southward shift is likely to occur, potentially leading to subpopulation differentiation. Forecasted range changes by different demographic classes suggest class-specific niches must be accounted for when predicting changes in species occurrence for developing conservation and management strategies that can effectively protect a species. Projecting marine species suitable habitats exclusively from temperature data could underestimate climate vulnerability, and additional variables describing a species habitat should also be included to obtain more accurate species distribution models (McHenry et al. 2019). Here, we provide further evidence that these types of projections can be significantly improved for marine predators if models also account for predicted shifts in their potential prey. Our framework is broadly applicable to other predator species and ecosystems, to more adequately anticipate where animals might be found in the future.

## Supplementary Information

Below is the link to the electronic supplementary material.

Supplementary file1 (PDF 2144 KB)

## Data Availability

The data used in this publication have been previously used by Fitzpatrick et al. (2012) and Holmes et al. (2014).
