# Correction to: Forecasting intraspecific changes in distribution of a wide-ranging marine predator under climate change

**DOI:** 10.1007/s00442-021-05092-6

**Published:** 2021-12-17

**Authors:** Yuri Niella, Paul Butcher, Bonnie Holmes, Adam Barnett, Robert Harcourt

**Affiliations:** 1 Department of Biological Sciences, Macquarie University, North Ryde, Sydney, NSW 2113 Australia; 2 NSW Department of Primary Industries, National Marine Science Centre, PO Box 4321, Coffs Harbour, NSW 2450 Australia; 3 Southern Cross University, National Marine Science Centre, PO Box 4321, Coffs Harbour, NSW 2450 Australia; 4 School of Science, Technology and Engineering, University of the Sunshine Coast, Sippy Downs, QLD 4556 Australia; 5 School of Biological Sciences, University of Queensland, St Lucia, Brisbane, QLD 4067 Australia; 6 College of Science and Engineering, James Cook University, Townsville, Australia; 7 Marine Data Technology Hub, James Cook University, Townsville, Australia; 8 Biopixel Oceans Foundation, Smithfield, Australia

## Correction to: Oecologia 10.1007/s00442-021-05075-7

The article “Forecasting intraspecific changes in distribution of a wide‑ranging marine predator under climate change”, written by Yuri Niella, Paul Butcher, Bonnie Holmes, Adam Barnett and Robert Harcourt was originally published electronically on the publisher’s internet portal on 17 November 2021 without open access. With the author(s)’ decision to opt for Open Choice the copyright of the article changed on 10 December 2021 to © The Author(s) 2021 and the article is forthwith distributed under a Creative Commons Attribution 4.0 International License, which permits use, sharing, adaptation, distribution and reproduction in any medium or format, as long as you give appropriate credit to the original author(s) and the source, provide a link to the Creative Commons licence, and indicate if changes were made. The images or other third party material in this article are included in the article’s Creative Commons licence, unless indicated otherwise in a credit line to the material. If material is not included in the article’s Creative Commons licence and your intended use is not permitted by statutory regulation or exceeds the permitted use, you will need to obtain permission directly from the copyright holder. To view a copy of this licence, visit http://creativecommons.org/licenses/by/4.0/

The original article has been corrected.

