# Supplementary material for: Forecasting intraspecific changes in distribution of a wide-ranging marine predator under climate change
Source: Oecologia. 2021 Nov 17;198(1):111–24. doi: 10.1007/s00442-021-05075-7 (PMC8803685; doi:10.1007/s00442-021-05075-7)
Supplement: Supplementary file 1 — Supplementary file1 (PDF 2144 KB) [file 442_2021_5075_MOESM1_ESM.pdf]

Forecasting intraspecific changes in distribution of a wide-ranging marine predator under climate change

Yuri Niella, Paul Butcher, Bonnie Holmes, Adam Barnett, Robert Harcourt

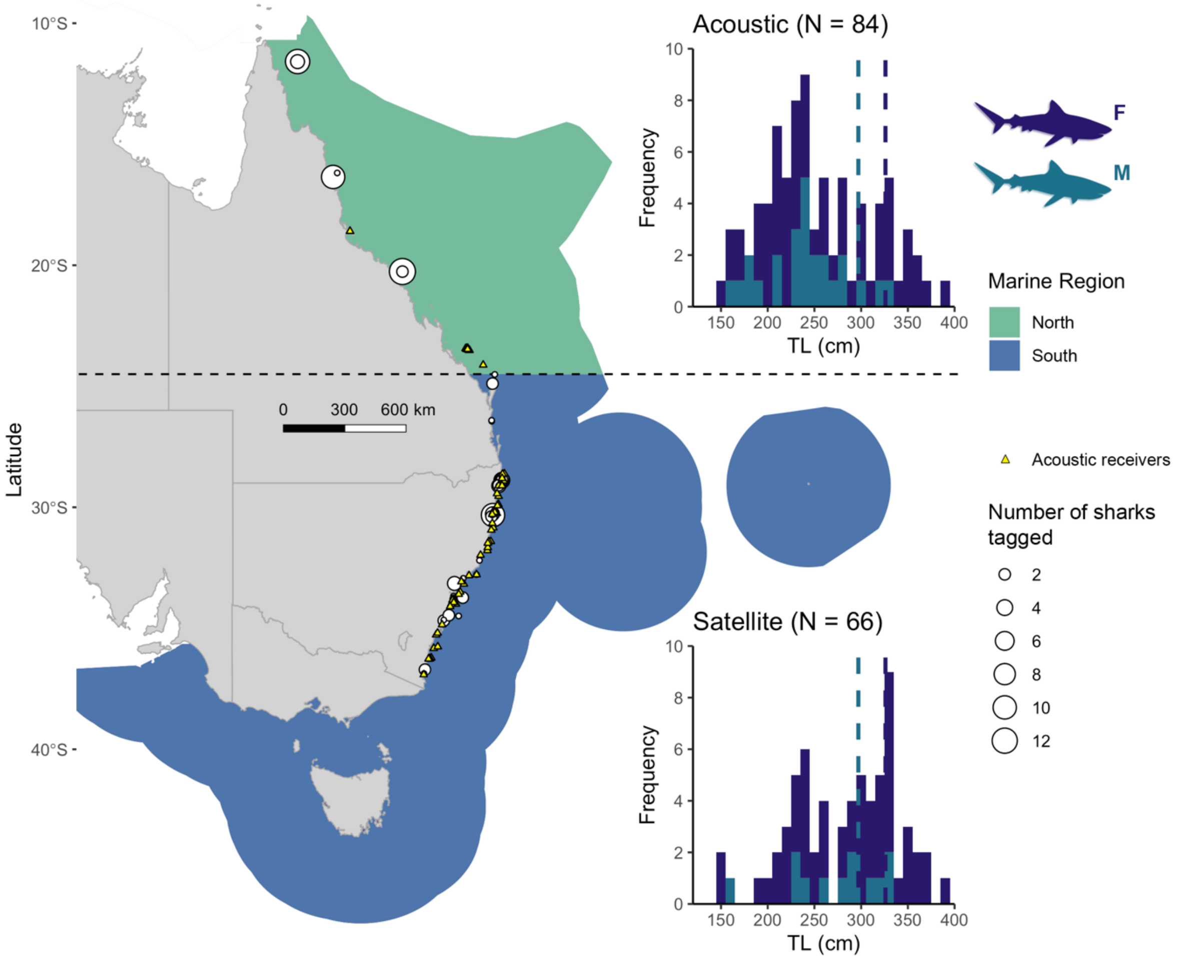

Figure S1. Map of the east coast of Australia showing the positions of acoustic receivers and the tiger shark tagging locations, including the North and South Marine Regions, and histograms of total length (TL) distribution of sharks tagged with acoustic (upper) and satellite (lower) transmitters by sex. Vertical dashed lines in histograms represent sex-specific maturation sizes for tiger sharks in eastern Australia. The horizontal dashed line represents the coast centroid location (latitude 24.5°S) used to divide the North and South Regions considered in the present study.

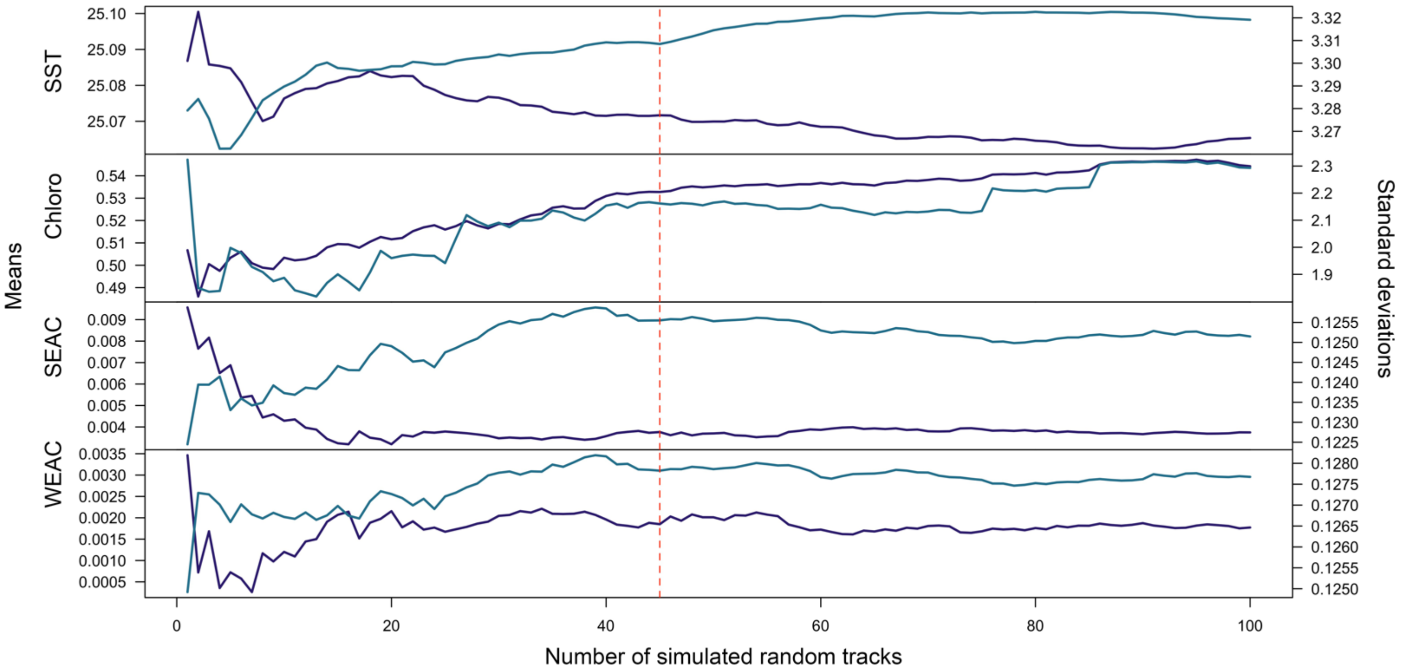

Figure S2. Variation of mean (dark line) and standard deviation (light line) of the environmental variables as a function of the number of simulated random tracks. The final value of 45 random tracks (dashed red line) was chosen as it stabilized all environmental variables.

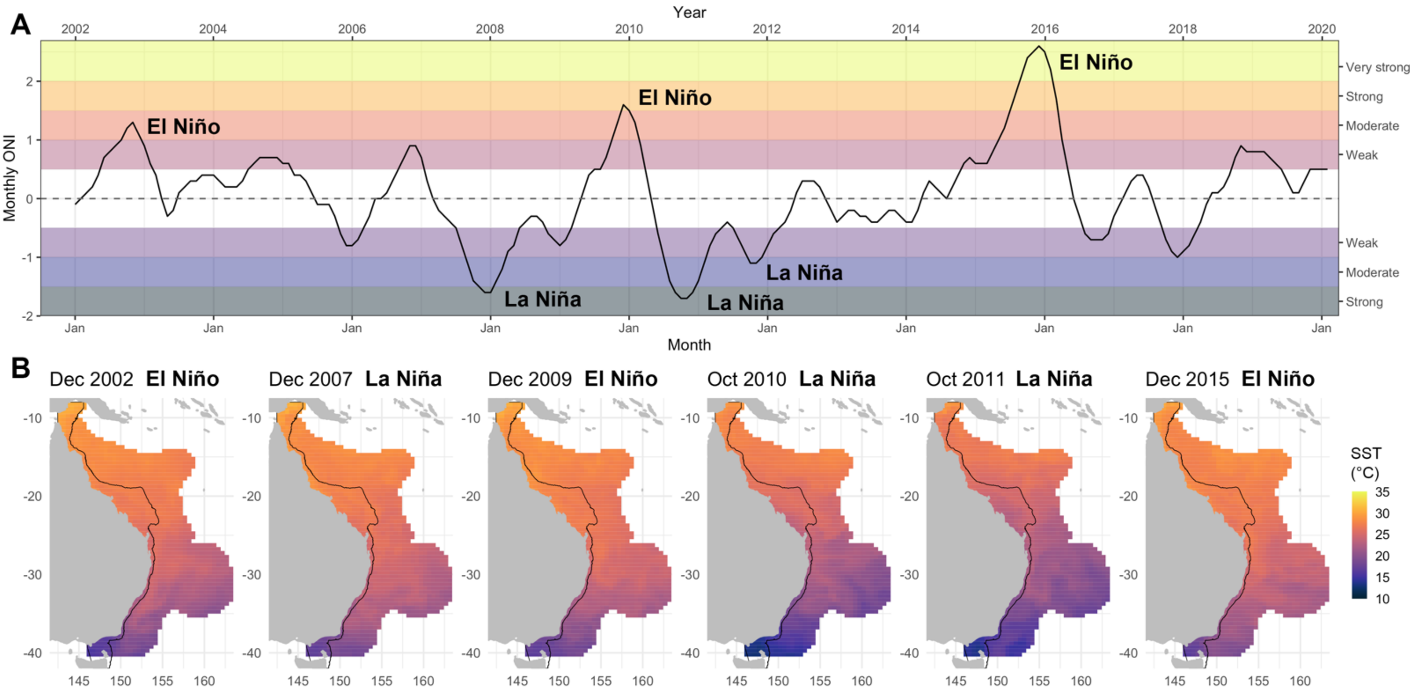

Figure S3. Monthly distribution (a) of the Oceanic Niño Index (ONI) with horizontal bands representing the corresponding intensity of events. Geographical distribution within the Australian marine regions (b) of the mean sea surface temperature (SST) during peaked El Niño/La Niña months during the study period, with contour lines depicting the 1,000-m isobath used as the spatial resolution for the modelling approach.

1. Shark location processing

GPE3 software (Wildlife Computers) was used to generate raw tracks from light-level data of PSAT tags, and a Kalman Filter was applied to the corresponding processed geolocations and location errors. A correlated random-walk state-space model using a 5 m/s speed filter to avoid unrealistic swimming behaviour was applied to both PSAT and SPOT data, with location estimates reduced to standard sampling intervals of 24 hours using the foieGras R package (Jonsen et al. 2020). A bathymetric correction was performed with locations over land moved to the nearest in-water location within the corresponding location errors. False detections were identified and excluded from the acoustic tracking dataset. These comprised either any acoustic detections of the tracked transmitters which occurred prior to the release of a tagged shark, or single/few detections which would correspond to biologically not plausible movements (e.g., hundreds to thousands of kilometres in one day/a couple of days).

One of the main differences between satellite and acoustic telemetry data is that in the latter the animal positions are restricted to the deployment locations of the acoustic receivers (Harcourt et al. 2019). To merge acoustic and satellite data and remove potential bias in the analysis from multiple daily detections of the same individual, the acoustic dataset was standardised to only include a single daily location for each tracked shark. These single daily acoustic locations were obtained using centre of activity calculations (Simpfendorfer et al. 2002). For double-tagged sharks, these processed acoustic locations were added to the satellite tracks. If the centre of activity location was placed within the error of a satellite position on a particular day, the acoustic position was used, and the satellite excluded as the latter are usually less accurate. If the acoustic location was outside of the satellite error, the midpoint of a straight line between the two positions was considered to representative that day's position.

2.1. Juvenile shark movements

2.1.1. Juvenile females

Sharks tagged in the Great Barrier Reef Marine Park tended to remain in the northern region though some individuals swum towards Papua New Guinea or the Solomon Islands, with the furthest movement a straight-line distance of 2,138 km from the Great Barrier Reef, October 2015 towards Tonga, February 2016 (Fig. 1a). Juvenile females tagged in the Temperate East Marine Region usually moved further, both into the northern region but also towards oceanic areas including to Papua New Guinea and New Caledonia (Fig. 1a).

2.1.2. Juvenile males

Juvenile males tagged in the Great Barrier Reef Marine Park remained north of the coast centroid, some moving towards Papua New Guinea (Fig. 1b). Juvenile males tagged in the Temperate East Marine Region generally travelled further, with one moving towards New Caledonia (Fig. 1b).

2.2. Adult shark movements

2.2.1. Adult females

Adult females tagged in the Great Barrier Reef Marine Park remained in the north (Fig. 1c). Most sharks remained within Australian coastal waters, but one individual moved into the Gulf of Carpentaria, and another travelled north towards Papua New Guinea (Fig. 1c). Adult females tagged in the Temperate East Marine Region generally dispersed further than sharks tagged north of the coast centroid and moved both north and south into the South East Marine Region, using waters as far south as the Bass Strait (Fig. 1c) between the months of February and April when SSTs ranged between 17.8 and 20.5°C.

Table S1. Tagging and tracking metadata on the 115 tiger sharks analysed in the present study, including information on tagging date, location, sex, total length (TL; cm), types of transmitters deployed (Acoustic, SPOT and PSAT), total number of tracking days, maximum distances travelled away from tagging locations, and biological class.

Date	Latitude	Longitude	Sex	TL	Acoustic	SPOT	PSAT	Days	Distance	Class
21/01/2012	-33.147	151.376	F	150	FALSE	TRUE	TRUE	26	730.71	Juvenile
06/04/2019	-36.708	149.996	F	150	TRUE	FALSE	FALSE	285	331.78	Juvenile
10/04/2010	-33.732	151.764	F	152	FALSE	TRUE	TRUE	13	205.34	Juvenile
16/09/2017	-30.320	153.150	F	159	TRUE	FALSE	FALSE	112	90.79	Juvenile
25/04/2019	-30.322	153.180	F	160	TRUE	FALSE	FALSE	40	140.85	Juvenile
07/03/2019	-36.718	149.992	F	168	TRUE	FALSE	FALSE	269	932.52	Juvenile
16/11/2017	-30.362	153.120	F	174	TRUE	FALSE	FALSE	12	171.88	Juvenile
13/02/2018	-30.287	153.203	F	188	TRUE	TRUE	TRUE	281	857.85	Juvenile
03/03/2018	-34.647	150.875	F	192	TRUE	FALSE	FALSE	10	133.70	Juvenile
09/09/2018	-28.885	153.595	F	192	TRUE	FALSE	FALSE	209	878.70	Juvenile
18/11/2007	-35.054	151.066	F	200	FALSE	TRUE	TRUE	47	459.54	Juvenile
15/04/2019	-29.096	153.444	F	201	TRUE	FALSE	FALSE	281	843.44	Juvenile
15/11/2017	-30.272	153.154	F	203	TRUE	FALSE	FALSE	4	165.06	Juvenile
15/04/2019	-29.107	153.450	F	203	TRUE	FALSE	FALSE	77	31.92	Juvenile
18/06/2018	-29.113	153.457	F	204	TRUE	FALSE	FALSE	137	122.35	Juvenile
27/04/2012	-34.492	151.572	F	210	FALSE	TRUE	FALSE	107	1118.01	Juvenile
12/02/2015	-16.353	145.697	F	210	TRUE	TRUE	FALSE	194	28.43	Juvenile
29/11/2017	-30.285	153.141	F	210	TRUE	FALSE	FALSE	324	715.33	Juvenile
10/01/2018	-30.271	153.158	F	212	TRUE	FALSE	FALSE	4	6.80	Juvenile
28/01/2018	-30.380	153.110	F	212	TRUE	FALSE	FALSE	56	144.53	Juvenile
02/03/2018	-34.684	150.876	F	212	TRUE	FALSE	FALSE	328	545.37	Juvenile
29/03/2019	-30.323	153.180	F	216	TRUE	TRUE	TRUE	298	895.84	Juvenile
09/02/2018	-32.195	152.548	F	219	TRUE	FALSE	FALSE	370	492.50	Juvenile
29/01/2018	-30.274	153.155	F	221	TRUE	FALSE	FALSE	7	95.45	Juvenile
30/01/2018	-30.318	153.153	F	221	TRUE	TRUE	FALSE	329	435.72	Juvenile
24/04/2019	-28.813	153.614	F	223	TRUE	TRUE	TRUE	26	356.08	Juvenile
23/01/2019	-28.855	153.613	F	226	TRUE	FALSE	TRUE	5	24.91	Juvenile
30/05/2017	-30.331	153.138	F	227	TRUE	TRUE	FALSE	948	788.07	Juvenile
17/05/2019	-29.112	153.457	F	227	TRUE	FALSE	FALSE	44	14.26	Juvenile
14/05/2019	-30.318	153.145	F	228	TRUE	TRUE	TRUE	200	559.29	Juvenile
19/11/2019	-33.798	151.303	F	230	TRUE	FALSE	FALSE	18	16.46	Juvenile
23/10/2018	-16.353	145.697	F	231	FALSE	TRUE	FALSE	476	105.40	Juvenile
08/05/2019	-30.237	153.185	F	236	TRUE	TRUE	TRUE	233	2394.83	Juvenile
22/10/2018	-16.353	145.697	F	240	FALSE	TRUE	FALSE	509	780.79	Juvenile
29/03/2017	-29.115	153.459	F	241	TRUE	FALSE	FALSE	182	60.99	Juvenile
13/02/2018	-30.318	153.190	F	242	TRUE	TRUE	TRUE	533	603.53	Juvenile
18/12/2018	-20.259	148.941	F	242	TRUE	TRUE	FALSE	247	322.78	Juvenile
27/03/2012	-34.459	151.114	F	245	FALSE	TRUE	TRUE	27	1001.29	Juvenile

08/04/2012	-34.816	151.102	F	250	FALSE	TRUE	FALSE	285	1262.34	Juvenile
29/03/2019	-30.315	153.173	F	251	TRUE	TRUE	TRUE	61	1029.18	Juvenile
24/07/2011	-24.512	153.264	F	260	FALSE	TRUE	TRUE	7	2.77	Juvenile
12/08/2015	-16.353	145.697	F	260	TRUE	TRUE	FALSE	84	103.61	Juvenile
06/11/2019	-32.936	151.790	F	260	TRUE	FALSE	FALSE	71	116.94	Juvenile
07/02/2019	-28.853	153.615	F	261	TRUE	FALSE	FALSE	161	236.55	Juvenile
23/10/2018	-16.353	145.697	F	262	FALSE	TRUE	FALSE	453	132.42	Juvenile
31/10/2018	-28.868	153.599	F	270	TRUE	FALSE	FALSE	114	26.45	Juvenile
12/08/2015	-16.353	145.697	F	280	TRUE	TRUE	FALSE	158	72.24	Juvenile
04/02/2019	-28.841	153.616	F	280	TRUE	FALSE	FALSE	174	604.10	Juvenile
12/06/2019	-20.259	148.941	F	285	TRUE	TRUE	FALSE	278	263.43	Juvenile
01/01/2019	-28.892	153.584	F	287	TRUE	FALSE	FALSE	140	877.56	Juvenile
25/03/2011	-26.444	153.121	F	290	FALSE	TRUE	FALSE	155	402.38	Juvenile
08/12/2006	-11.583	144.033	F	295	FALSE	TRUE	FALSE	63	169.34	Juvenile
16/12/2007	-11.588	144.031	F	296	FALSE	TRUE	TRUE	232	88.76	Juvenile
13/12/2007	-11.588	144.031	F	300	FALSE	TRUE	TRUE	21	451.27	Juvenile
18/02/2015	-16.353	145.697	F	300	TRUE	TRUE	FALSE	1300	2031.83	Juvenile
12/09/2019	-20.259	148.941	F	300	TRUE	TRUE	FALSE	186	338.60	Juvenile
04/02/2015	-24.890	153.159	F	301	TRUE	TRUE	FALSE	388	167.89	Juvenile
01/12/2013	-11.588	144.031	F	308	FALSE	TRUE	FALSE	16	149.38	Juvenile
26/09/2019	-28.884	153.585	F	308	TRUE	FALSE	FALSE	41	597.21	Juvenile
25/09/2010	-26.408	153.116	F	310	FALSE	TRUE	TRUE	10	194.44	Juvenile
25/03/2013	-11.588	144.031	F	311	FALSE	TRUE	FALSE	383	210.84	Juvenile
18/02/2015	-16.353	145.697	F	317	TRUE	TRUE	FALSE	385	2137.84	Juvenile
21/02/2002	-11.600	144.057	F	320	FALSE	TRUE	TRUE	90	473.82	Juvenile
05/05/2007	-34.290	151.637	F	320	FALSE	TRUE	TRUE	48	1838.04	Juvenile
27/03/2019	-36.871	149.950	F	320	TRUE	FALSE	FALSE	64	666.38	Juvenile
13/12/2019	-20.259	148.941	F	321	TRUE	TRUE	FALSE	70	1491.15	Juvenile
18/12/2002	-11.583	144.050	F	327	FALSE	TRUE	TRUE	79	106.80	Adult
03/12/2006	-11.583	144.033	F	330	FALSE	TRUE	FALSE	44	435.61	Adult
18/02/2015	-16.353	145.697	F	330	TRUE	TRUE	FALSE	472	650.00	Adult
23/07/2019	-28.873	153.605	F	330	TRUE	TRUE	TRUE	246	1350.26	Adult
13/09/2019	-20.259	148.941	F	330	TRUE	TRUE	FALSE	183	379.79	Adult
26/02/2020	-28.868	153.599	F	330	TRUE	FALSE	FALSE	786	600.33	Adult
25/03/2013	-11.588	144.031	F	331	FALSE	TRUE	FALSE	110	212.63	Adult
05/04/2008	-32.783	152.412	F	335	FALSE	TRUE	TRUE	1480	998.43	Adult
16/12/2018	-20.259	148.941	F	340	TRUE	TRUE	FALSE	425	550.92	Adult
23/11/2004	-11.583	144.033	F	350	FALSE	TRUE	FALSE	17	276.00	Adult
26/11/2006	-11.500	144.200	F	350	FALSE	TRUE	TRUE	43	67.33	Adult
09/05/2019	-33.671	151.331	F	350	TRUE	FALSE	FALSE	240	285.80	Adult
11/06/2019	-28.883	153.595	F	354	TRUE	FALSE	TRUE	199	747.44	Adult
02/02/2015	-24.890	153.159	F	355	TRUE	TRUE	FALSE	1580	1019.47	Adult
14/02/2015	-16.186	145.889	F	360	TRUE	TRUE	FALSE	203	64.80	Adult

06/01/2020	-20.263	148.942	F	360	FALSE	TRUE	FALSE	65	115.41	Adult
13/07/2019	-29.107	153.452	F	364	TRUE	FALSE	FALSE	15	260.03	Adult
26/11/2007	-11.588	144.031	F	368	FALSE	TRUE	TRUE	121	301.42	Adult
18/12/2018	-20.259	148.941	F	370	TRUE	TRUE	FALSE	453	1164.02	Adult
10/06/2019	-20.259	148.941	F	386	TRUE	TRUE	FALSE	276	230.30	Adult
08/05/2019	-30.322	153.180	M	157	TRUE	TRUE	TRUE	46	1418.17	Juvenile
25/05/2019	-29.091	153.445	M	167	TRUE	FALSE	FALSE	30	15.50	Juvenile
09/05/2019	-30.237	153.187	M	176	TRUE	FALSE	FALSE	192	9.93	Juvenile
12/01/2018	-30.386	153.105	M	182	TRUE	FALSE	FALSE	651	287.53	Juvenile
07/02/2018	-34.697	150.871	M	186	TRUE	FALSE	FALSE	20	150.58	Juvenile
06/01/2018	-28.816	153.616	M	212	TRUE	FALSE	FALSE	296	335.87	Juvenile
15/02/2018	-28.871	153.607	M	215	TRUE	FALSE	FALSE	284	154.40	Juvenile
18/02/2015	-16.353	145.697	M	230	TRUE	TRUE	FALSE	159	762.61	Juvenile
16/12/2018	-20.259	148.941	M	230	TRUE	TRUE	FALSE	228	79.45	Juvenile
23/01/2019	-29.105	153.446	M	234	TRUE	FALSE	TRUE	2	126.98	Juvenile
20/03/2019	-28.778	153.606	M	239	TRUE	FALSE	FALSE	217	36.45	Juvenile
11/04/2019	-33.742	151.335	M	240	TRUE	FALSE	FALSE	78	607.25	Juvenile
15/04/2018	-34.657	150.876	M	242	TRUE	FALSE	FALSE	426	186.73	Juvenile
11/12/2019	-20.259	148.941	M	245	TRUE	TRUE	FALSE	94	274.32	Juvenile
13/12/2019	-28.848	153.611	M	245	TRUE	FALSE	FALSE	2	2.59	Juvenile
25/01/2019	-30.323	153.179	M	248	TRUE	FALSE	TRUE	31	140.94	Juvenile
16/03/2019	-28.822	153.617	M	254	TRUE	FALSE	FALSE	28	31.53	Juvenile
15/03/2019	-29.088	153.446	M	263	TRUE	FALSE	FALSE	113	124.99	Juvenile
10/06/2019	-20.259	148.941	M	264	TRUE	TRUE	FALSE	205	163.82	Juvenile
20/02/2019	-28.839	153.617	M	266	TRUE	FALSE	FALSE	27	617.74	Juvenile
22/12/2018	-28.843	153.617	M	282	TRUE	FALSE	FALSE	39	760.16	Juvenile
09/05/2019	-30.328	153.152	M	284	TRUE	TRUE	TRUE	139	1196.19	Juvenile
18/12/2005	-11.589	144.031	M	288	FALSE	TRUE	FALSE	356	927.99	Juvenile
10/12/2007	-11.588	144.031	M	292	FALSE	TRUE	TRUE	409	241.69	Juvenile
06/11/2019	-33.780	151.301	M	300	TRUE	FALSE	FALSE	35	15.95	Adult*
17/04/2007	-33.569	151.370	M	310	FALSE	TRUE	TRUE	15	699.12	Adult*
10/06/2019	-20.259	148.941	M	316	TRUE	TRUE	FALSE	242	1021.49	Adult*
07/01/2020	-20.263	148.942	M	330	FALSE	TRUE	FALSE	69	701.15	Adult*
11/06/2019	-20.259	148.941	M	335	TRUE	TRUE	FALSE	221	360.31	Adult*

* Adult male sharks excluded from the analysis due to low sample size.

Table S2. Details of the response and explanatory variables used in the tiger shark modelling approach. The coast centroid has been delimited at latitude 24.5°S, as it corresponds to the current boundary between the northern Great Barrier Reef Marine Park and the Coral Sea marine regions, and the southern Temperate East marine region (please see Figure 1 for further details). All explanatory variables were modelled as candidate effects interacting with the three significant biological classes (1 = juvenile females; 2 = juvenile males; 3 = adult females).

Variable type	Variable name	Acronym	Details
Response	Location latitude		The observed track latitude including both acoustic and satellite positions of the tiger sharks monitored.
	Shark movement patterns in the North Region (latitude >24.5°S)		Daily binomial values including 0 for the simulated track locations and 1 for the locations from the observed shark satellite tracks.
	Shark movement patterns in the South Region (latitude ≦24.5°S)		Daily binomial values including 0 for the simulated track locations and 1 for the locations from the observed shark satellite tracks.
	Maximum dive depth		Shark daily maximum dive depths (in metres) recorded by Pop-up Satellite Archival Tags (PSAT).
Explanatory	Month	Month	Calendar month ranging from January (1) to December (12).
	Oceanic Niño Index	ONI	Monthly values corresponding to 3-month temperature differences calculated for the Niño 3.4 region (from 5°N, 120°W to 5°S 170°W) and is represented by a negative (cooling = La Niña) to positive (warming = El Niño) scale.
	Derivative Sea Surface Temperature	derSST	Difference between present and past 6-day average local SST (°C), with 0.5° latitude x 0.5° longitude resolution.
	Sea Surface Temperature difference from the 22°C isotherm	SST.22	Difference between present and past 6-day average local SST (°C) subtracted from the 22°C isotherm, with 0.5° latitude x 0.5° longitude resolution.
	Derivative chlorophyll-a	derChloro	Difference between present and past 6-day average local surface concentration of chlorophyll-a (mg/m^3), with 0.5° latitude x 0.5° longitude resolution.
	Region		Categorical variable representing the Marine Region (i.e. North or South Region) where a tiger shark track was located, based on the respective location latitude in relation to the 24°S latitude centroid.

Modelling tiger shark dispersal

Table S3. Stepwise variable selection procedure for the Generalized Additive Mixed Model of tiger shark latitude along the east coast of Australia. Included are the corresponding model Akaike Information Criterion (AIC), ΔAIC (model AIC - lowest AIC), and calculated AIC weights ($_{w}$AIC). The final selected model is highlighted in bold.

Variable	AIC	ΔAIC	$_{w}$AIC
Null	45952.28	7846.62	<0.0001
Class x Month	45191.67	7086.01	<0.0001
Class x ONI	44681.10	6575.44	<0.0001
Class x derSST	44430.00	6324.34	<0.0001
Class x SST.22	43483.45	5377.79	<0.0001
Class x SST.22 + Class x Month	39851.60	1745.94	<0.0001
Class x SST.22 + Class x ONI	41943.46	3837.80	<0.0001
Class x SST.22 + Class x derSST	42944.24	4838.58	<0.0001
Class x SST.22 + Class x Month + Class x ONI	39058.03	952.37	<0.0001
Class x SST.22 + Class x Month + Class x derSST	38880.02	774.36	<0.0001
**Class x SST.22 + Class x Month + Class x derSST + Class x ONI**	**38105.66**	**0.00**	**0.9999**

Modelling tiger shark movement patterns

Table S4. Stepwise variable selection procedure for the Generalized Additive Mixed Model of tiger shark habitat preference in the North Region. Included are the corresponding model Akaike Information Criterion (AIC), ΔAIC (model AIC - lower AIC), and calculated AIC weights ($_{w}$AIC). The final selected model is highlighted in bold.

Variable	AIC	ΔAIC	$_{w}$AIC
Null	79910.24	5182.47	<0.0001
Class x Month	79859.83	5132.06	<0.0001
Class x SST.22	77818.23	3090.46	<0.0001
**Class x derChloro**	**74727.77**	**0.00**	**0.9999**
Class x ONI	78976.56	4248.79	<0.0001
Class x derChloro + Class x Month*			
Class x derChloro + Class x SST.22*			
Class x derChloro + Class x ONI*			

* Model discarded due to lack of statistical significance

Table S5. Stepwise variable selection procedure for the Generalized Additive Mixed Model of tiger shark habitat preference in the South Region. Included are the corresponding model Akaike Information Criterion (AIC), ΔAIC (model AIC - lowest AIC), and calculated AIC weights ($_{w}$AIC). The final selected model is highlighted in bold.

Variable	AIC	ΔAIC	$_{w}$AIC
Null	16574.76	583.18	<0.0001
Class x Month	16497.55	505.97	<0.0001
**Class x SST.22**	**16107.63**	**0.00**	**0.9999**
Class x ONI	16516.79	525.21	<0.0001
Class x SST.22 + Class x Month*			
Class x SST.22 + Class x ONI*			

* Model discarded due to lack of statistical significance

Modelling tiger shark dive depth

Table S6. Stepwise variable selection procedure for the Generalized Additive Mixed Model of tiger shark maximum dive depth. Included are the corresponding model Akaike Information Criterion (AIC), ΔAIC (model AIC - lowest AIC), and calculated AIC weights ($_{w}$AIC). The final selected model is highlighted in bold.

Variable	AIC	ΔAIC	$_{w}$AIC
Null	10380.45	1028.16	<0.0001
Region x Month	10333.22	980.93	<0.0001
Region x ONI	9406.27	53.98	<0.0001
**Region x ONI + Region x Month**	**9352.29**	**0.00**	**0.9999**

* Model discarded due to lack of statistical significance

Class-specific distribution models

Table S7. List of potential prey occurrence data downloaded from the Ocean Biodiversity Information System (OBIS) for the period between January 2002 and December 2020.

Potential prey group	Common name	Scientific name	Number of records	Latitude range (min l max)
Sea turtles	Loggerhead turtle	*Caretta caretta*	130	-41.23 l -9.50
	Green turtle	*Chelonia mydas*	21,570	-38.78 l -9.40
	Hawksbill turtle	*Eretmochelys imbricata*	246	-42.02 l -8.10
	Leatherback turtle	*Dermochelys coriacea*	132	-41.50 l -8.50
	Olive Ridley turtle	*Lepidochelys olivacea*	346	-40.81 l -10.44
Crabs	Blue swimming crab	*Portunus pelagicus*	267	-37.75 l -9.82
	Three-spot swimming crab	*Portunus sanguinolentus*	147	-34.25 l -12.03
	Mud crab	*Scylla serrata*	421	-37.75 l -21.74
Dugongs	Dugong	*Dugong dugon*	5,241	-36.89 l -9.18
Sea snakes	Olive sea snake	*Aipysurus laevis*	363	-25.26 l -12.96
Birds	Silver gull	*Chroicocephalus novaehollandiae*	164,678	-44.54 l -9.24
	Brown booby	*Sula leucogaster*	2,407	-43.14 l -9.29
	Australian pelican	*Pelecanus conspicillatus*	6,505	-42.96 l -10.79
Teleosts	Smooth toadfish	*Tetractenos glaber*	114	-43.30 l -28.54
	Ocean puffer	*Lagocephalus lagocephalus*	607	-33.84 l -10.61
	Dusky flathead	*Platycephalus fuscus*	13,944	-38.25 l -27.03
Elasmobranchs	Spotted eagle ray	*Aetobatus ocellatus*	154	-22.74 l -9.51
	Eastern shovelnose ray	*Aptychotrema rostrata*	192	-36.22 l -22.03
	Smooth stingray	*Bathytoshia brevicaudata*	104	-43.30 l -32.20

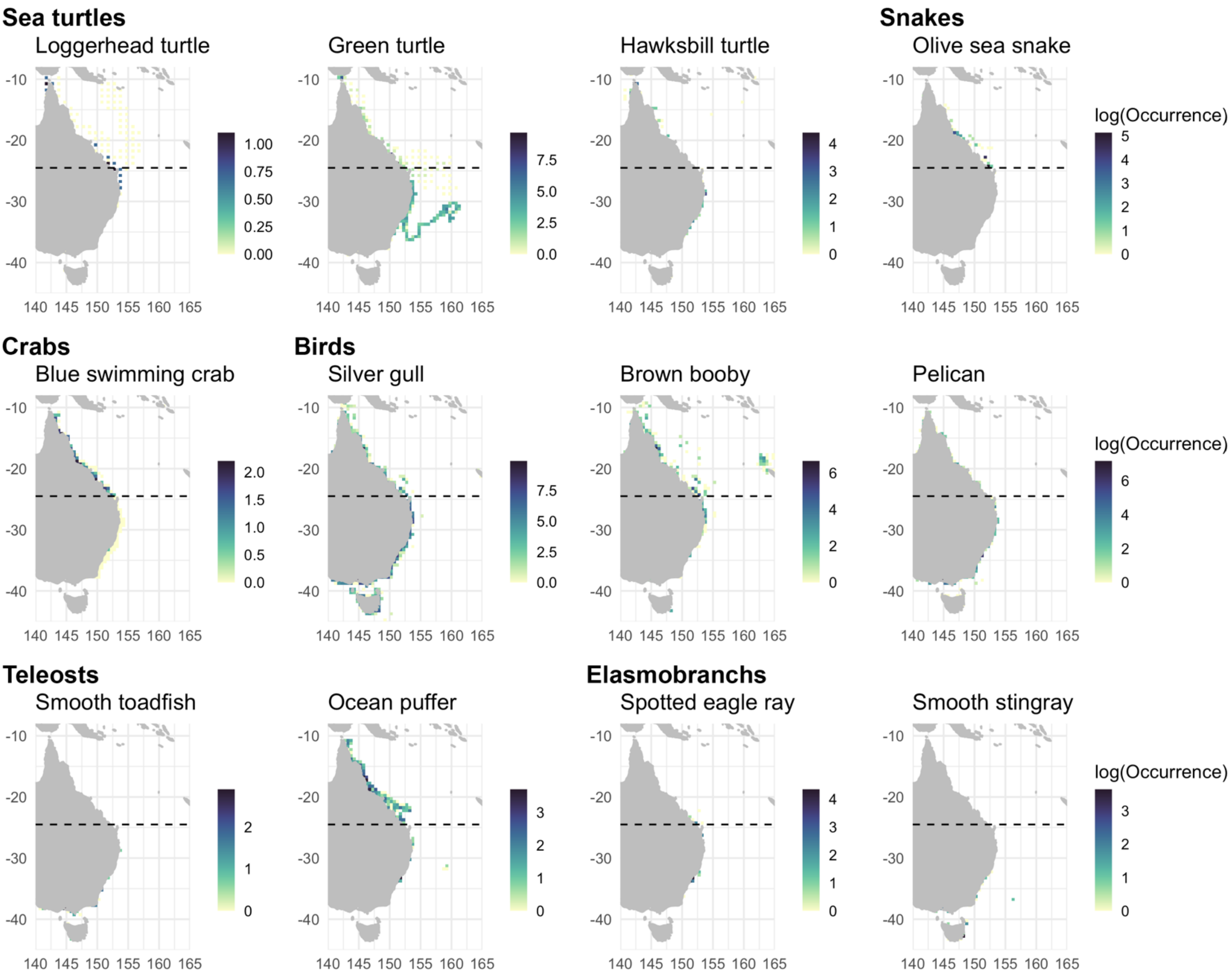

Figure S4. Species-specific occurrences between January 2002 and December 2020, downloaded from the Ocean Biodiversity Information System for tiger shark potential prey species (sea turtles, snakes, crabs, birds, teleosts and elasmobranchs) found to be positively correlated with tiger shark presence (Table S8).

Table S8. Generalized Linear Models of tiger shark occurrence as a function of potential prey abundance, including the significant positively correlated species for each shark biological class. Included are the coefficient estimates (Est), standard errors (SE), z-value (z) and p-values (p) of each model variable.

Biological class	Potential prey group	Common name	Est	SE	z	p
Juvenile Females	Sea turtles	Loggerhead	0.58	0.10	5.59	<0.001
		Green	0.29	0.01	23.25	<0.001
	Snakes	Olive sea snake	0.18	0.04	4.07	<0.001
	Crabs	Blue swimming	0.23	0.04	5.55	<0.001
	Birds	Gull	0.36	0.01	48.06	<0.001
		Booby	0.08	0.01	5.81	<0.001
		Pelican	0.06	0.01	4.52	<0.001
	Teleosts	Toadfish	0.17	0.04	4.11	<0.001
		Puffer	0.63	0.02	25.81	<0.001
	Elasmobranchs	Smooth stingray	0.21	0.09	2.28	0.022
Juvenile Males	Sea turtles	Green	0.47	0.02	21.25	<0.001
	Birds	Gull	0.19	0.02	10.89	<0.001
	Teleosts	Puffer	1.39	0.04	33.91	<0.001
	Elasmobranchs	Spotted eagle ray	0.64	0.16	4.08	<0.001
Adult Females	Sea turtles	Loggerhead	1.28	0.10	12.49	<0.001
		Green	0.24	0.02	13.44	<0.001
		Hawksbill	0.26	0.06	4.16	<0.001
	Snakes	Olive sea snake	0.14	0.04	3.36	<0.001
	Birds	Gull	0.27	0.01	23.04	<0.001
		Pelican	0.32	0.02	17.23	<0.001
	Teleosts	Puffer	0.95	0.03	29.38	<0.001

Table S9. Generalized Additive Models of thermal influence upon the species-specific occurrences of tiger shark potential prey. Included are the effective degrees of freedom (edf), reference degrees of freedom (Ref.df), F-value (F) and p-value (p), from the respective modelled effects of sea surface temperature for each species.

Potential prey group	Common name	edf	Ref.df	F	p
Sea turtles	Loggerhead	3.60	3.91	5.27	<0.001
	Green	3.46	3.84	20.86	<0.001
	Hawksbill*	1.87	2.30	1.20	0.386
Snakes	Olive sea snake*	3.23	3.68	2.08	0.051
Crabs	Blue swimming	2.37	2.87	8.32	<0.001
Birds	Gull	2.77	3.29	4.80	0.001
	Booby	3.27	3.71	5.79	<0.001
	Pelican	1.00	1.00	12.55	<0.001
Teleosts	Toadfish	2.74	3.25	11.95	<0.001
	Puffer	3.68	3.94	10.76	<0.001
Elasmobranchs	Smooth stingray	3.11	3.59	3.69	0.022
	Spotted eagle ray*	1.00	1.00	1.12	0.291

* Non-significant species removed from the analysis.
